# Molecular age prediction using skull bone samples from individuals with and without signs of decomposition: a multivariate approach combining analysis of posttranslational protein modifications and DNA methylation

**DOI:** 10.1007/s00414-024-03314-z

**Published:** 2024-09-11

**Authors:** J. Becker, V. Bühren, L. Schmelzer, A. Reckert, S. B. Eickhoff, S. Ritz, J. Naue

**Affiliations:** 1 Institute of Legal Medicine, University Hospital Duesseldorf, 40225 Duesseldorf, Germany; 2 Institute of Forensic Medicine, Medical Center - University of Freiburg, Faculty of Medicine, University of Freiburg, 79104 Freiburg, Germany; 3 Institute for Systems Neuroscience, University Hospital Duesseldorf, 40225 Duesseldorf, Germany; 4 Institute of Neuroscience and Medicine, Brain & Behaviour (INM-7), Research Centre Juelich, 52428 Juelich, Germany

**Keywords:** Chronological age prediction, D-aspartic acid, Pentosidine, DNA methylation, Bone, Multivariate models

## Abstract

The prediction of the chronological age of a deceased individual at time of death can provide important information in case of unidentified bodies. The methodological possibilities in these cases depend on the availability of tissues, whereby bones are preserved for a long time due to their mineralization under normal environmental conditions. Age-dependent changes in DNA methylation (DNAm) as well as the accumulation of pentosidine (Pen) and D-aspartic acid (D-Asp) could be useful molecular markers for age prediction. A combination of such molecular clocks into one age prediction model seems favorable to minimize inter- and intra-individual variation. We therefore developed (I) age prediction models based on the three molecular clocks, (II) examined the improvement of age prediction by combination, and (III) investigated if samples with signs of decomposition can also be examined using these three molecular clocks. Skull bone from deceased individuals was collected to obtain a training dataset (*n* = 86), and two independent test sets (without signs of decomposition: *n* = 44, with signs of decomposition: *n* = 48). DNAm of 6 CpG sites in *ELOVL2*,* KLF14*,* PDE4C*,* RPA2*,* TRIM59* and *ZYG11A* was analyzed using massive parallel sequencing (MPS). The D-Asp and Pen contents were analyzed by high performance liquid chromatography (HPLC). Age prediction models based on ridge regression were developed resulting in mean absolute errors (MAEs)/root mean square errors (RMSE) of 5.5years /6.6 years (DNAm), 7.7 years /9.3 years (Pen) and 11.7 years /14.6 years (D-Asp) in the test set. Unsurprisingly, a general lower accuracy for the DNAm, D-Asp, and Pen models was observed in samples from decomposed bodies (MAE: 7.4–11.8 years, RMSE: 10.4–15.4 years). This reduced accuracy could be caused by multiple factors with different impact on each molecular clock. To acknowledge general changes due to decomposition, a pilot model for a possible age prediction based on the decomposed samples as training set improved the accuracy evaluated by leave-one-out-cross validation (MAE: 6.6–12 years, RMSE: 8.1–15.9 years). The combination of all three molecular age clocks did reveal comparable MAE and RMSE results to the pure analysis of the DNA methylation for the test set without signs of decomposition. However, an improvement by the combination of all three clocks was possible for the decomposed samples, reducing especially the deviation in case of outliers in samples with very high decomposition and low DNA content. The results demonstrate the general potential in a combined analysis of different molecular clocks in specific cases.

**Supplementary Information:**

The online version contains supplementary material available at 10.1007/s00414-024-03314-z.

## Introduction

Postmortem chronological age prediction of an individual can be crucial in determining the identity of a deceased. Methodological options for age prediction depend on the extent of postmortem changes and the availability of tissues; the initial situation can range from the presence of a complete corpse to the presence of only some pieces of bone. Teeth and bones are the most resistant human tissues that can withstand harsh conditions such as degradation and putrefaction due to their high content of inorganic substance [1]. Therefore, they are the most relevant sample types for forensic practice in these cases.

Conventional methods for chronological age prediction are based on the examination of physiological and degenerative changes (especially in dental and skeletal structures) during life [2]. However, in adulthood the accuracy of these methods may be low and cannot be used when morphological information is limited, e.g., in cases with only body parts or fragments of bones [2]. In the last decade, numerous new approaches based on the use of known age-dependent molecular changes have expanded the repertoire of age prediction methods [3]. Among the most interesting approaches for forensic age prediction are DNA methylation (DNAm) and post-translational protein modifications, such as accumulation of D-aspartic acid (D-Asp), and pentosidine (Pen) in long-living proteins.

Skull bones, like other bones in the body, are composed of both inorganic and organic tissue. The bone matrix consists of around 35% organic and 65% inorganic constituents. The inorganic components mainly include hydroxyapatite crystals, but also potassium, chlorine, iron, magnesium and carbonate [4, 5]. These crystals provide rigidity and hardness to bone tissue, contributing to its compressive strength [5]. The organic matrix of bone consists mainly of collagen fibers (approx. 90%), which provide tensile strength and flexibility to bone tissue. The remaining components are non-collagenous proteins, such as osteonectin, osteocalcin, sialoprotein, phosphoproteins, glycoproteins, proteoglycans, albumin and others [4]. Different processes of bone protein modifications can result from enzymatic or non-enzymatic processes [5, 6]. The enzymatic process involves the activation of lysyl oxidase, leading to the formation of immature and mature crosslinks that stabilize the collagen fibrils. Two types of non-enzymatic processes result in an accumulation of advanced glycation end products and D-aspartic acid in bone proteins. These bone protein modifications are age-related and can affect the mechanical properties of bone [5].

The accumulation of D-Asp with age is the result of spontaneous nonenzymatic conversion of L-asparagine and L-aspartic acid into its D-forms (for details, see [7]). Age prediction based on the D-Asp content in dentine, a very stable and bradytrophic tissue, revealed accurate estimates of 2.19–2.93 years mean absolute errors (MAE) [8–13]. This approach also works for more complex and heterogenous tissues with higher turnover, such as bone. However, for other tissues than dentine, the accuracy of the method is significantly lower, as long as suitable proteins are not purified and protein mixtures are analyzed (for details see [3]). Pen is an advanced glycation end product that accumulates as a fluorescent crosslink between arginine and lysine in different proteins like collagen [14]. Pathological metabolic conditions, such as long-lasting hyperglycemic states or renal failure, may result in elevated Pen levels [14]. Nevertheless, the analysis of Pen can be used for age estimation in cases in which confounding factors can be excluded or as an additional parameter, e.g. in combination with D-Asp [15].

Various cell types within the bone matrix are present to maintain bone structure, function, and repair. Osteoblasts are responsible for producing the organic matrix that serves as the foundation for mineral deposition and therefore are vital for growth and repair, ensuring the integrity and resilience of bone tissue. Derived from osteoblasts, osteocytes nestled within the bone matrix, monitor remodeling processes, and respond to mechanical stresses, playing a crucial role in maintaining bone metabolism [16]. In contrast, osteoclasts are involved in bone resorption by breaking down old or damaged bone tissue, releasing vital calcium and phosphate ions into the circulation to maintain mineral balance [17]. To allow these specific functions, epigenetic mechanisms such as DNAm play a role in the regulation of developmental processes, differentiation, and function in bone cells, too [18–20]. In addition to these primary cells, mesenchymal stem cells in the bone marrow serve tissue regeneration purposes, and endothelial cells form the intricate network of blood vessels within bones and assist in remodeling and repair processes [16]. Blood cells can also be seen as part of the overall cell type composition of bones. Studies revealed age-related DNAm alterations in all cells and tissues including bones [21, 22]. These modifications can influence gene expression patterns and contribute to genomic instability and the onset and progression of numerous diseases [23–27].

Various age-related DNAm markers, with increased or decreased methylation at specific cytosine-guanine (CpG) sites, have already been identified [21]. Based on these findings, several mathematical models for chronological age prediction in forensic settings have been proposed for various tissues and body fluids, including teeth and bone, obtaining MAE of 3–5 years (for details see reviews [28–30]).

Each of these parameters suggested for molecular age prediction exhibits limitations due to its specific biological context, including tissue-specific differences, individual (stochastic) changes, and many endogenous and exogenous factors, which could influence the degree of DNAm and accumulation of D-Asp and Pen, respectively [14, 30–32]. The combined analysis of all three molecular clocks addresses different biological levels and, therefore may compensate for the effects of various influencing factors on the accuracy of age estimation, especially in adulthood. Approaches combining multiple biological molecular clocks have already been tested and partially improved age prediction accuracy [33–36]. Data from a pilot study performing a parallel analysis of D-Asp, Pen, and DNAm revealed age-dependent changes in bone tissue (skull) and thereby indicated a potential for age prediction [37]. Within that study, the regions *ELOVL2*,* KLF14*,* PDE4C*,* RPA2*,* TRIM59*, and *ZYG11A*, showed a high correlation with age in bone (ρ 0.9–0.98). The age dependency of these markers was also seen in bone samples examined in other studies, e.g. [38, 39].

Within this study, we analyzed D-Asp and Pen as well as DNAm at multiple CpG sites in the six selected regions, in skull samples from deceased individuals without and with signs of decomposition. We developed and evaluated ridge regression models for age prediction and investigated the impact of postmortem changes on age prediction accuracy in samples ranging from early to advanced stages of decomposition.

## Materials and methods

### Actions taken to avoid contamination

The samples were treated with appropriate measures from collection to analysis (contamination protection through appropriate rooms, gloves, masks, gowns and clean preparation equipment, ‘human DNA-free’ tubes, pipette tips and reagents). The surfaces of the bones were cleaned with Biocidal and appropriate negative controls were carried out from the DNA extraction onwards as well as for the HPLC to ensure that no contamination had occurred as a result of the washing protocol.

### Bone sample collection and preparation

Samples of skull bone (Os parietale) were collected from 190 individuals during autopsy (0 to 96 years, 55 females and 135 males), sampling a piece from the left parietal bone, close to the usual saw cut for skull opening. In order to avoid heat exposure and because of the extremely small thickness of the calotte in the sampling area, the cancellous bone between the tabula externa and interna was not removed, so that the samples covered the entire bone cross-section. Sample information can be found in Suppl. Table S1A. The state of decomposition (d-score) was defined during autopsy by forensic pathologists based on morphological characteristics. The decomposition scores of head, trunk and extremities were evaluated (details in Suppl. Table S1B) and total body decomposition scores were calculated by summarizing the scores of those regions leading to a minimum score of 3 for corpses without any signs of decomposition and to a maximum score of 22 in highly decomposed bodies as described by Megyesi et al. [40]. Dataset 1 (*n* = 98) and dataset 2 (*n* = 44) contain only samples from individuals without signs of decomposition. Dataset 3 (*n* = 48) consists of all samples with early to advanced signs of decomposition. In all datasets, collection of samples from individuals over the whole age range was anticipated. However that was limited especially for individuals below 18 and for decomposed bodies. The processing of all three datasets was done independently to allow the use of split datasets for model training and independent testing (see below). Soft tissue was mechanically removed with a sterile scalpel and bone samples were sliced into approx. 1 × 1 × 0.5 cm large fragments. The samples were pulverized with a tube mill at 17.000 rpm (Ika Tube Mill Control, Staufen, Germany). The resulting powder was washed in distilled water, 15% sodium chloride, 2% sodium dodecyl sulfate, and ethanol/ether (vol. 3:1), respectively, lyophilized by a freeze-drying system (Christ, Osterode am Harz, Germany) and stored at -80 °C until further analysis.

### Determination of the D-Asp content by analysis of D- and L-aspartic acid

From each sample, 1 mg of bone powder was hydrolyzed for 6 h with 1 mL of 6 N HCl at 100 °C. D-aspartic acid and L-aspartic acid were analyzed by high-performance liquid chromatography (HPLC; 1100 Series and 1260 Infinity II, Agilent, CA, USA) as described by Becker et al. [33] with minor modification (shortened gradient: 47 min). Samples were dissolved in 1 mL sample buffer (0.01 M HCl with 1.5 mM sodium azide and 0.03 mM L-homo-arginine). For HPLC analysis, a C18 column (Hypersil BDS C18, 250 × 3 mm, particle size 5 μm, Thermo Fisher Scientific (TFS), Waltham, MA, USA) was used as stationary phase. The mobile phase included eluents A (23 mM sodium acetate, 1.5 mM sodium azide, and 1 mM EDTA) and B (92.3% methanol, 7.7% acetonitrile). The amino acid enantiomers were detected by a gradient over a period of 49 min at a constant flow rate of 0.56 mL/min. Amino acids were detected at an excitation wavelength of λ = 230 nm and a detection wavelength of λ = 445 nm. D- aspartic acid (D) and L-aspartic acid (L) were identified by their retention times. The total content of D-Asp was expressed as ln((1 + D/L)/(1 – D/L)).

### Determination of pentosidine content

20 mg of bone powder per sample were hydrolyzed with 1 mL of 6 N HCl at 110 °C for 18 h. After drying, 1 mL of 0.01 M heptafluorobutyric acid (HFBA) was added. The solution was filtered through syringe filters (Ø 25 mm and 0.45 μm pore diameter). The dried samples were dissolved in 200 µL of 0.01 M HFBA. Analyzes were performed by HPLC (1100 Series). The stationary phase was a semi-preparative column (Onyx™ Monolithic SemiPrep C18, 100 × 4.6 mm, Phenomenex, Torrance, CA, USA). The mobile phase consisted of eluents A (40 mM NaH_2_PO_4_, 1.5 mM sodium azide, 0.1% HFBA, pH = 2.70) and B (45% methanol, 45% acetonitrile, 10% H_2_O) based on Heems et al. [41]. A total of 10 µL of each sample was injected into the HPLC system. Samples were detected over a period of 28 min using a linear gradient followed by a washing plateau (100% eluent B) of 6 min. The flow rate was constant at 1 mL/min and the column temperature at 40 °C. Pentosidine (Pen) was measured at an excitation wavelength of λ = 335 nm and a detection wavelength of λ = 385 nm. The signal of Pen was identified by its retention time. The Pen content was expressed as (area of Pen [ - ])/(bone powder [mg])

### Analysis of DNA methylation

#### DNA extraction and bisulfite conversion

Per sample up to 150 mg of bone powder were decalcified and lysed according to the Supplementary Protocol from Qiagen: Extraction of DNA from bone or teeth using the EZ1 DNA Investigator Kit (June 2016; Qiagen, Hilden, Germany). DNA extraction was performed according to the “Large-Volume Protocol” of the EZ1 DNA Investigator Kit and the EZ1 Advanced XL extraction robot (Qiagen). DNA was eluted in 100 µL elution buffer. DNA was quantified using the PowerQuant System (Promega, Madion, WI, USA). The quantity of DNA for further analysis was assessed on the longer 294 bp fragment, and DNA quality by evaluation of degradation (84 bp short fragment/ 294 bp long fragment). 200 ng of DNA (when possible) were bisulfite converted using single column and 96well plate-based EZ DNA Methylation Gold Kits (Zymo Research Europe, Freiburg, Germany). DNA was eluted in 15 µL DNA-free water and roughly quantified using the ssDNA Quantification Kit and Qubit 2.0 and Flex (both TFS).

#### Marker amplification and massive parallel sequencing

Parts of the genomic regions *ELOVL2*,* KLF14*,* PDE4C*,* RPA2*,* TRIM59* and *ZYG11A* were amplified in a multiplex approach and information on genomic position, primer sequences, and concentrations can be found in the Suppl. Table S2. PCR was performed using 7.5 µL PyroMark Master Mix (Qiagen), 1.5 µL Coral Load (Qiagen), 0.5 µg BSA (TFS), 1.5 µL of the multiplex primer mix, 4µL of bisulfite converted DNA, and water ad 15 µL. Cycling was carried under the following conditions: 10 min at 95 ºC; 15 cycles: 45 s at 98 ºC, 30 s at 54 ºC, 30 s at 72 ºC; 25 cycles: 45 s at 98 ºC, 30 s at 62 ºC, 30 s at 72 ºC; final elongation for 10 min at 72 ºC on the MJ Research PTC-200 (BioRad, Hercules, CA, USA). PCR products were cleaned using 1.9x magnetic beads (GE Healthcare, Little Chalfont, UK), which were prepared according to [42]. PCR for adapter addition was carried out in a 12 µL-volume using 1 µL of PCR product, 6 µL NEBNext Ultra II Q5 Master Mix (New England Biolabs, Ipswich, MA, USA), 5 pmol of Nextera XT i5- and i7 index primer each and 2.25 µL of DNA-free water under the following conditions: 30 s at 98 ºC; 6 cycles: 10 s at 98 ºC, 30 s at 62 ºC, 45 s at 65 ºC; final elongation for 5 min at 65 ºC. PCR products were cleaned twice using 1.6x of the prepared magnetic beads and then quantified using the dsDNA high-sensitivity Qubit Quantification Kit (TFS). PCR products were equimolar pooled and the final 11pM library 2 × 150 bp sequenced on a MiSeq FGx (Verogen, San Diego, CA, USA) using the micro, nano, and ‘normal size’ 300 bp v2 kits (Illumina, San Diego, CA, USA).

The FastQ files were quality checked and 5′ and 3′ trimmed (TrimGalore v0.4.3 [43] (including the FastQC package)). The paired-end reads were merged (PEAR v0.9.10 [44]) and aligned to the human reference hg19 (samtools implemented in the biscuit v0.2.2 package [45]). CpG as well as non-CpG (i.e. CHH and CHG) DNA methylation (DNAm) values were extracted to obtain the DNAm values at the age-dependent positions as well as to check the bisulfite conversion efficiency (MethylDackel v0.2.1 [46]). The anticipated minimal coverage (merged reads) of 1000 was obtained for all samples, except one sample of a decomposed bone with a coverage of 600 merged reads for all markers.

### Data analysis and statistical evaluation

Data analysis and visualization was performed using JupyterLab 3.4.4 (Anaconda Navigator v2.3.1) with Python 3.9 and the analysis packages pandas v1.4.4, pingouin v0.5.2, seaborn v0.11.2. The relationship between chronological age and the accumulation of D-Asp, Pen and DNAm was tested by rank correlation, and the corresponding Spearman correlation coefficients (ρ) were determined. For outlier detection, the Mahalanobis distance was determined to detect outliers for the different CpG positions, Pen, as well as D-Asp outside the expected chi² distribution (cutoff: 0.95).

### Development of age prediction models

Development and evaluation of the age prediction models was done using julearn 0.3.1 with pandas 2.1.4, sklearn 1.3.2, numpy 1.24.4 and scipy 1.9.1 was used for development and evaluation of the age prediction models.

### Development and evaluation of the age prediction models

Age prediction ridge regression models that included the different molecular markers individually (DNAm, D-Asp, Pen) as well as combinations (D-Asp + Pen, D-Asp + DNAm, Pen + DNAm, and D-Asp + Pen + DNAm) were built on a training set with individuals of dataset 1 equal to or above 18 years (*n* = 86, 18 to 96 years) and z-score pre-processing. In the first step, the best alpha value (L2 penalty) was determined using hyperparameter tuning (5-fold cross-validation (CV)). The final value of alpha = 5 was chosen for all feature combinations. 10-times 10-fold CV was used for development and first evaluation of model performance. The final model for the 86 training samples was evaluated in an independent test set (*n* = 44, 19 to 96 years). An additional evaluation was performed using samples with signs of decomposition (*n* = 48, 20 to 90 years). Model creation using julearn is based on the implementation of the sklearn function run_cross_validation and directly includes the CV approach. The mean absolute errors (MAE), root mean square errors (RMSE), and R value were determined for a model evaluation.

### Development and evaluation of a pilot age prediction model specific for decomposed samples

To obtain first insights, if the development of an age prediction model using decomposed samples for training, and therefore including the heterogeneity of post-mortem effects, could be beneficial, a pilot ridge regression model was developed and evaluated with leave-one-out CV (LOOCV) using dataset 3 (decomposed samples, *n* = 48).

## Results

### Correlation of D-Asp, Pen and DNAm markers with age in skull bone samples

**Fig. 1:**
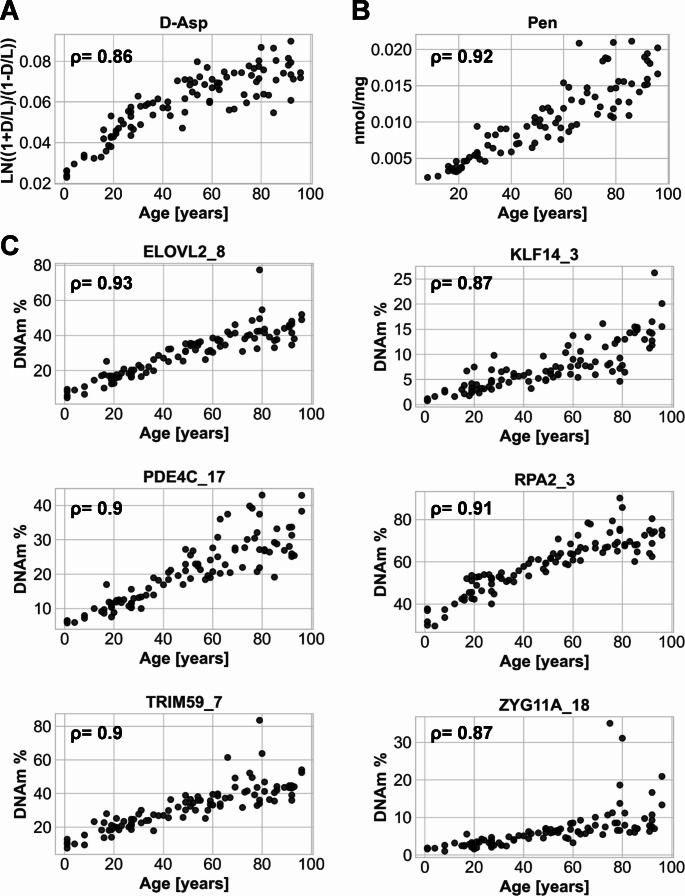
Results of the selected age-dependent protein and DNA methylation markers in training data (*n* = 98; cf. Suppl. Table S1A). Accumulation of D-Asp (**A**) and Pen (**B**). In case of Pen, values are missing due to detection limits for most of the under 15 years old. (**C**) DNA methylation levels for the final selected CpG sites from six amplicon regions. ρ: Spearman‘s rho. D: D-Asp, L: L-Asp

The samples from dataset 1 (*n* = 98, without signs of decomposition) were analyzed for total D-Asp (described as LN((1 + D/L)/(1-D/L))), Pen (nmol/mg) and the DNAm (%) from the regions *ELOVL2*, *KLF14*, *PDE4C*, *RPA2*, *TRIM59*, and *ZYG11A* (Fig. 1).

As expected from the pilot study, an accumulation of Pen and D-Asp with increasing age was observed in bone samples, resulting in Spearman’s ρ-D-Asp = 0.86, 95%, CI: 0.79–0.9, and ρPen = 0.92, 95% CI: 0.79–0.9 in dataset 1. In the case of eight samples of very young individuals, Pen determination was not possible as the Pen accumulation did not reach the analytical threshold. The Spearman’s ρ was also calculated for the CpG positions analyzed, leading to the selection of the final CpG positions within the amplicon for further analysis: ELOVL2_8 (ρ = 0.93, 95% CI: 0.79–0.9), KLF14_3 (ρ = 0.87, 95% CI: 0.81–0.91) PDE4C_17 (ρ = 0.9, 95% CI: 0.85–0.93), RPA2_3 (ρ = 0.91, 95% CI: 0.87–0.94), TRIM59_7 (ρ = 0.9, 95% CI: 0.86–0.93), and ZYG11A_18 (ρ = 0.87, 95% CI: 0.82–0.91). Also, other CpG positions in the same amplicons showed ρ-values above 0.8 and could also be useful for age prediction (data not shown). Applying the Mahalanobsis distance at a chi^2^ level of 0.95, revealed data points with a higher divergence from the center point (Suppl. Fig. S1). All deviating samples (in 17 individuals), except one (4 years, RPA2_3) were over 60 years old with four individuals having deviating samples at least at three markers. Most of the samples with greater divergence were detected in PDE4C_17 (*n* = 7). However, the occurrence of single outliers can increase in case of markers with a very high age correlation, as lower inter-individual differences can lead to more values identified as outlier.

### Independent datasets with samples from individuals without and with signs of decomposition

**Fig. 2:**
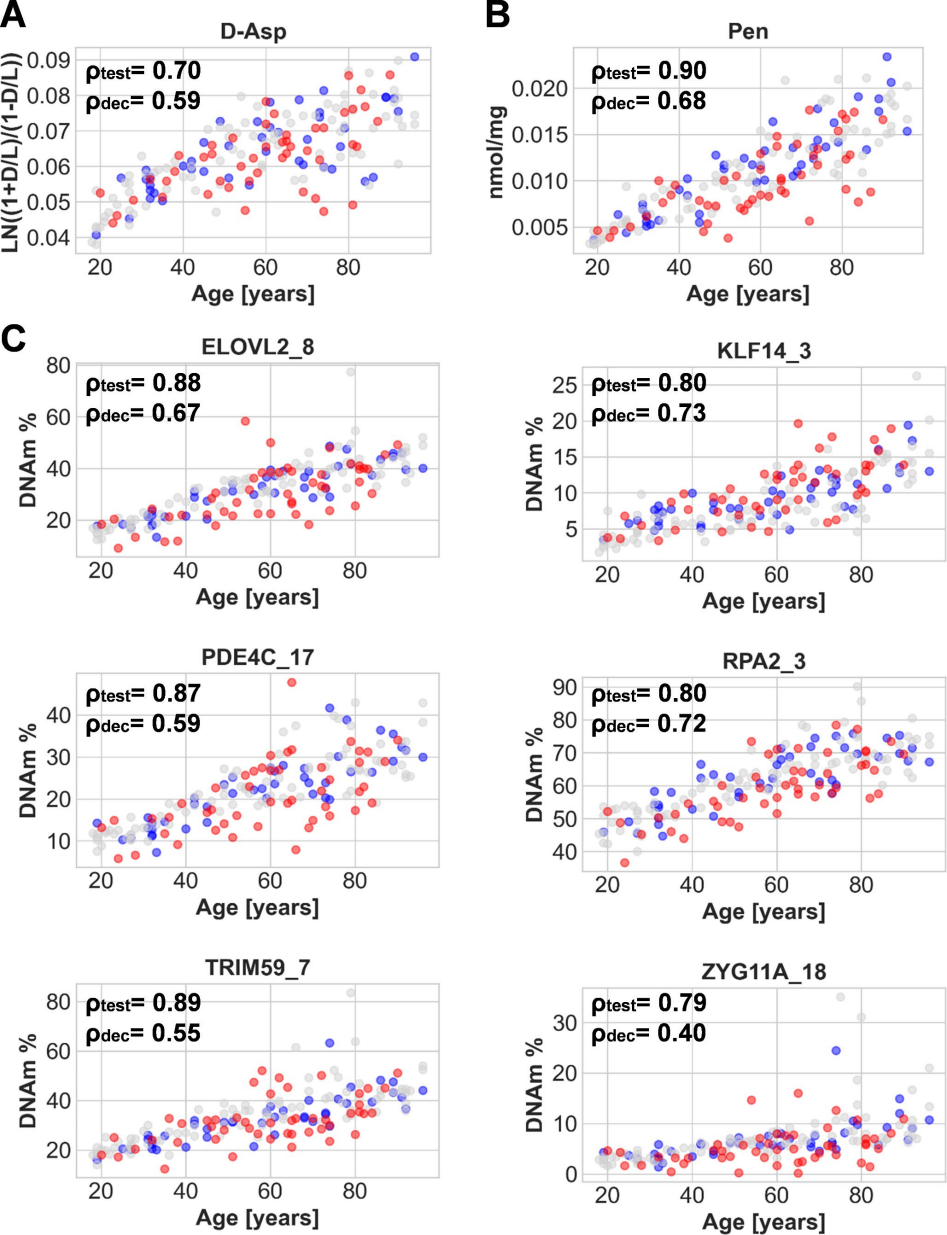
Results of the selected age-dependent protein and DNA methylation markers. The training data (Dataset 1 with age > = 18 (grey, *n* = 86)), independent data (dataset 2, samples without signs of decomposition (blue, *n* = 44) and dataset 3, samples with signs of decomposition (red, *n* = 48)). Accumulation of D-Asp (**A**) and Pen (**B**). DNA methylation levels for the final selected CpG sites from six amplicon regions (**C**). ρtest: Spearman‘s rho dataset 2 (no signs of decomposition), ρdec: Spearman‘s rho dataset 3 (decomposed samples). D: D-Asp, L: L-Asp, DNAm: DNA methylation

In addition to bone samples from individuals without signs of decomposition on which an age prediction model will be build (see section below), two additional datasets were collected for further evaluation. An independent dataset 2 (*n* = 44) with samples from individuals without signs of decomposition was collected to verify the observed age-dependent changes (Fig. 2). Additionally, 48 samples from individuals with low to strong decomposition (cf. staging in Material and Methods) were included in the study to determine whether and how age-dependent changes are also reflected in these challenging samples (dataset 3). The observed accumulation of Pen and DAsp was detectable in the test dataset (dataset 2) as in decomposed samples (dataset 3); however, with differences in the degree of correlation (test dataset: ρ = 0.90 (Pen), ρ = 0.70 (DAsp); decomposed dataset: ρ = 0.68 (Pen), ρ = 0.59 (D-Asp)) (Fig. 2). The lower ρ values are due to a higher scattering of the values for both parameters increasing substantially with age with a downward trend for some samples. In the case of DNAm, the same trends were observed. The age dependence was verified for all six markers with the test data (ρ = 0.79–0.89) and the decomposed samples (ρ = 0.4–0.73). Here, higher variability was observed for the older individuals and the decomposed samples, too. The downward trend was less distinct, but also visible except for KLF14.

### Age prediction models based on training data and cross validation

#### Composition of training data set and hyperparameter optimization for model development

As Pen evaluation was difficult for samples under 18 years (no results in eight cases) and to avoid bias in the development of machine models buildings due to the restricted sample numbers for individuals below age, only individuals equal or above 18 years from dataset 1 were included in the training data set. Therefore, 86 samples were finally included for model(s) development. Ridge regression (RR), a type of linear regression, was chosen as underlying algorithm for the age prediction model, as it is a suitable model in case of a possible multicollinearity (correlation between the independent used markers) and avoiding overfitting by inclusion of a regularization term (penalty term alpha -L2). In the first step, hyperparameter optimization was conducted to determine the best L2 (alpha) value for each model by CV analysis. As the value did not show a strong impact and was always close to five, the final alpha = 5 was chosen for all RR models.

#### Single-molecular clock models

RR models (10times 10-fold CV) for D-Asp, Pen, and DNAm were built and evaluated (based on the CV) on the 86 training data samples. The resulting mean MAEs and RMSEs can be found in Table 1. The best age prediction results were obtained for the DNAm marker set (MAE: 4.95 years, RMSE: 6.89 years). Protein marker-based RR models revealed MAEs/ RMSEs of 9.66 years/ 11.52 years (Pen) and 11.91 years/14.47 years (D-Asp). As expected due to observed values and known accumulating inter-individual differences with increasing age, the MAE and RMSE show different accuracies considering only prediction results within specific age groups (Suppl. Table S3).

**Table 1:** MAEs and RMSEs for training data (age ≥ 18 of dataset 1), test data (dataset 2) and individuals with signs of decomposition (dataset 3). The results are based on 10times 10-fold CV ridge regression model development and evaluation for D-Asp, Pen and DNAm, as well as combinations. CV = cross validation, MAE = Mean absolute error, RMSE = root mean square error, CI = confidence interval, D-Asp = accumulation of D-aspartic acid, Pen = accumulation of pentosidine, DNAm = DNA methylation

Marker combination	10-times 10xCV Training data (dataset 1, *n* = 86)		Individuals without signs of decomposition Test data (dataset 2, *n* = 44)		Individuals with signs of decomposition (dataset 3, *n* = 48)	
	Mean MAE CV (± 95% CI) [years]	Mean RMSE CV (± 95% CI) [years]	MAE [years]	RMSE [years]	MAE [years]	RMSE [years]
D-Asp	11.91 (11.3–12.5)	14.57 (13.9–15.3)	11.72	14.57	11.68	15.42
Pen	9.66 (9.3–10.0)	11.52 (11.1–12.0)	7.72	9.26	11.77	15.07
DNAm	4.95 (4.6–5.3)	6.89 (6.4–7.4)	5.48	6.56	7.38	10.39
D-Asp + Pen	8.55 (8.2–8.9)	10.18 (9.7–10.6)	7.16	9.16	10.81	14.08
D-Asp + DNAm	4.86 (4.5–5.2)	6.81 (6.3–7.3)	5.39	6.5	7.07	9.81
Pen + DNAm	4.91 (4.6–5.2)	6.72 (6.2–7.2)	5.1	6.36	7.02	9.39
D-Asp + Pen + DNAm	4.93 (4.6–5.2)	6.63 (6.1–7.1)	5.11	6.34	6.8	9.08

#### Combined molecular clocks

In addition, four combinations of the three molecular clocks were used for the construction and evaluation of the RR models (Table 1). Especially, the combination of the Pen and D-Asp markers in one RR model showed an improvement on the overall MAE (8.55 years) and RMSE (10.18 years), while the combination of the protein markers with the DNAm markers did not reveal a general advantage (MAE/RMSE 4.86 years/ 6.81 years (D-Asp + DNAm), 4.91 years/ 6.72 years (Pen + DNAm), 4.93 years/ 6.63 years (DAsp + Pen + DNAm) within the CV-evaluated data. However, it has to be considered that the DNAm analysis includes six markers that enter into the RR model as individual independent markers and therefore have an overall higher impact on the model. As for the single molecular clock RR models, higher MAEs and RMSEs were obtained with increasing age (Suppl. Table S3).

#### Age prediction of independent samples from individuals with and without signs of decomposition

**Fig. 3:**
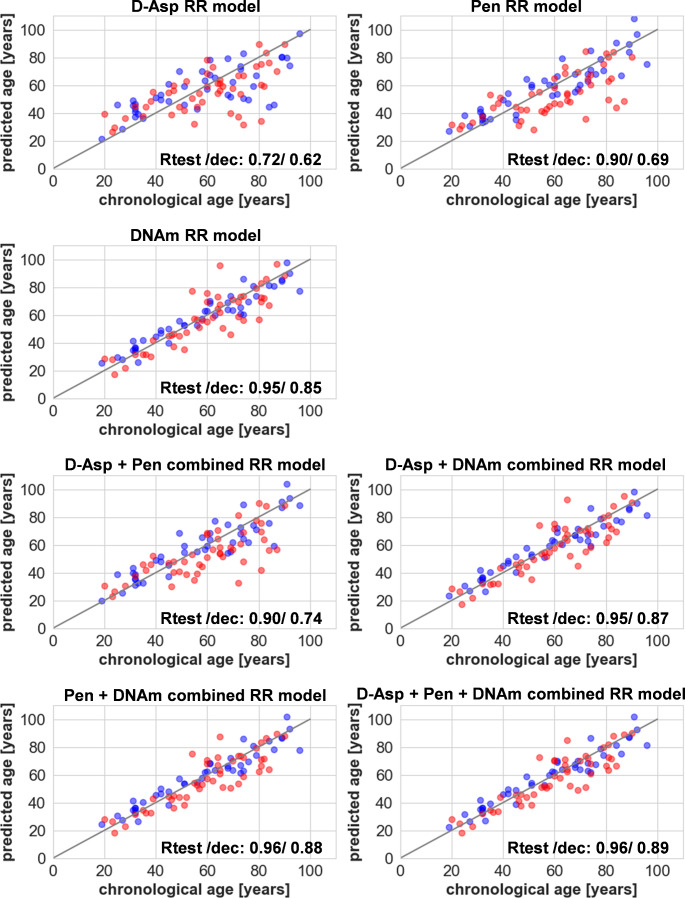
Age predictions results for individuals of dataset 2 (individuals without signs of decomposition (blue)), and dataset 3 (individuals with signs of decomposition (red)). The RR models were developed with the training set. cf. Table 1. Rtest: dataset 2 (no signs of decomposition), dec: dataset 3 (with signs of decomposition); RR = ridge regression

The individual and combined RR models were tested on the independent datasets using, as in the training dataset, individuals ≥ 18 years (dataset 2: *n* = 44, individuals without signs of decomposition and dataset 3: *n* = 48, individuals with signs of decomposition). The MAE and RMSE results obtained of dataset 2 are comparable to the CV evaluation (Table 1; Fig. 3), with even slightly better results for Pen and D-Asp. This could be explained by small differences in the composition of the datasets and single outlier samples. The age prediction of dataset 3 samples revealed an overall lower prediction accuracy, except for D-Asp. Accuracy was especially decreased by the strong underestimation of age in a subset of samples. (Fig. 3).

#### Deeper investigation of samples from individuals with signs of decomposition

The state of decomposition of the individual from which the bone sample originates could influence the age accuracy achieved via a prediction model. Suppl. Fig. S2A shows all prediction results of the single molecular clocks and of the overall combined RR model in dependence on the d-score. No systematic directional shift to under- or over-prediction dependent on the decomposition was observed (Suppl. Fig. S2B). Also, no statistically significant correlation was found between the absolute prediction error and the dscore for the RR models based on DNAm (ρ = 0.2, *p* = 0.30), Pen (ρ = 0.005, *p* = 0.97), D-Asp (ρ=-0.2, *p* = 0.30), and the combined RR model DNAm + Pen + D-Asp (ρ = 0.16, *p* = 0.38) (Suppl. Fig. S2C).

A comparison of the results of each single-molecular age prediction RR model (DNAm, Pen, and D-Asp), and the DNAm + DAsp + Pen combined RR model was performed for the total body dscore (Suppl. Fig. S3A, C, E) and head-specific d-score (Suppl. Fig. S3B, D, F). The calculation of the difference of the absolute prediction error for each sample between the single RR models (DNAm, Pen, DAsp, respectively) and the combined model for total body d-score as well as head-specific d-score revealed that the combination was partially able to improve the age prediction.

As for individuals without signs of decomposition, the highest improvement in prediction accuracy for individuals with signs of decomposition was obtained in the combined model compared to individual D-Asp and Pen RR models (Suppl. Fig. S3A-D). In case of DNAm, in which the sole DNAm RR model revealed a similar overall accuracy to the combined model, a deeper analysis was performed to investigate if the combination could improve prediction in single cases. In 13 of 48 samples, an improvement on the age prediction accuracy (at least 2 years better prediction) was observed by the marker combination. However, the use of the combined model also reduced the accuracy in case of 8 samples (Suppl. Fig. S3E, F). Due to the lower values of D-Asp and Pen in a subset of samples in 6 of 8 samples, the decrease in accuracy and a stronger underestimation of the age than in the case of the DNAm-based age prediction model are explicable. Yet, the combination with the proteins generally does not lead to a stronger underestimation compared to the DNAm RR model. In 7 of the 13 cases with improved prediction, inclusion of the protein parameters increased the calculated age, leading to a lower absolute error. For the two protein clocks, an improvement was obtained by the combination of all molecular clocks for 34 (Pen) and 30 (D-Asp) samples out of 48 samples, and a decrease in accuracy was obtained in 9 (Pen) and 11 (D-Asp) samples (Suppl. Fig S3A-D). Overall, the amount (years) of improvement was higher compared to the amount of decrease.

However, investigating the results in dependence on the d-score on a single case basis rather than a systematic improvement or bias, especially in the case of samples with an overall high total body d-score greater than 15 or head-specific d-score greater than 6, an improvement was visible for the combined RR model compared to the DNAm RR model. Within this study, no limit for a minimal amount of DNA was set for further processing to allow better research on the variation observed in challenging samples. Unsurprisingly, a higher variation in the deviations between chronological and predicted age can be attributed to the lower DNA content. In these cases, the combination (or sole) analysis of the protein markers was beneficial (Suppl. Fig. S4A-C).

For the proteins, improvement and decrease were not associated with specific d-score ranges. Considering the d-score, and the comparison of the D-Asp RR model and combined RR model, a slight tendency to less improvement was seen by combination of the molecular clocks for very strongly decomposed individuals (Suppl. Fig. S3A, B). Considering that the DNAm + Pen + D-Asp RR model improved age prediction for samples with very low DNA content compared to the DNAm RR model, it could be assumed that the Pen RR model or D-Asp RR model could even be more advantageous without additional inclusion of DNA in these cases. However, as can be seen in Suppl. Figures S4A, B, no clear conclusion can be drawn from the samples within this study, as the addition of the DNAm analysis was still advantageous in roughly half of the cases with very low DNA content compared to a pure D-Asp RR model or Pen RR model.

**Fig. 4:**
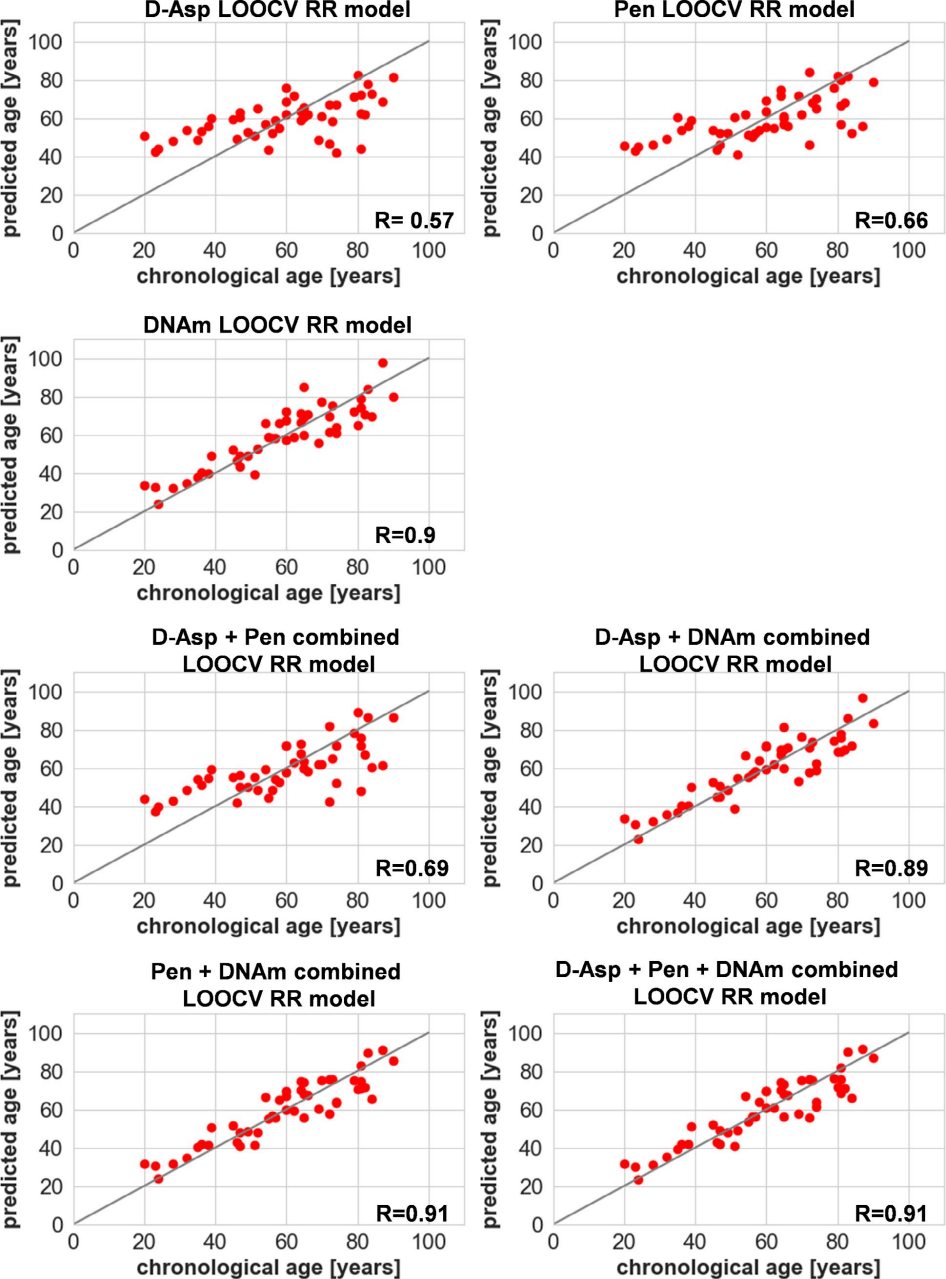
LOOCV ridge regression model for dataset 3 (decomposed samples). Results of LOOCV age predictions for the different marker combinations used for model building, *n* = 48, cf. Table 2; LOOCV = leave-one-out cross validation, RR = ridge regression

We further investigated, if an RR model developed with samples from individuals with signs of decomposition as training data might optimize the overserved decreased accuracy by inclusion of the variation due to decomposition. As the number of decomposed samples was restricted in this study, a leave-one-out CV (LOOCV) approach was chosen for evaluation (Fig. 4; Table 2, Suppl. Table S3) and should be considered as a pilot model with need of further research. The developed RR model specifically trained with samples of individuals with signs of decomposition led to an improvement in overall accuracy compared to the predictions based on the model trained on samples from individuals without signs of decomposition (cf. Figure 3; Table 1).

**Table 2:** MAE and RMSE results of the leave-one-out-CV model trained with samples of individuals with signs of decomposition. LOOCV = leave-one-out-cross validation, MAE = Mean absolute error, RMSE = Root Mean Square Error, D-Asp = accumulation of D-aspartic acid, Pen = accumulation of pentosidine, DNAm = DNA methylation

Marker combination	Individuals with signs of decomposition LOOCV (*n* = 48)	
	MAE [years]	RMSE [years]
D-Asp	11.94	14.88
Pen	10.88	13.67
DNAm	6.56	8.09
D-Asp + Pen	10.4	13.07
D-Asp + DNAm	6.44	8.1
Pen + DNAm	6.11	7.39
D-Asp + Pen + DNAm	6.19	7.61

## Discussion

The methodological possibilities for chronological age prediction of a deceased person depend on the availability of the biological material. Skull bone is among the most commonly found bone type and agedependent accumulation of molecular markers like Pen and D-Asp as well as changes in the DNAm level could therefore be useful for age prediction [37]. As interindividual variations are known for all chronological age markers [47, 48], a combined analysis could be beneficial. For this purpose, we investigated age-dependent changes in these three molecular clocks, developed age prediction RR models, investigated the improvement of age prediction by combination, and examined whether samples from individuals with signs of decomposition can also be analyzed. To achieve this, parietal bones from deceased individuals without and with signs of decomposition were collected and the DNAm of 6 CpG sites in *ELOVL2*, *KLF14*, *PDE4C*, *RPA2*, *TRIM59*, and *ZYG11A* was analyzed using MPS, and the amount of D-Asp and Pen was determined by HPLC. To the best of our knowledge, this is the first study to examine age prediction using DNAm, D-Asp and Pen for samples in different stages of decomposition in bone.

### Age-dependent changes in D-Asp, Pen and DNA methylation

The samples included in dataset 1 (*n* = 98) were used to characterize the age-dependent molecular clocks D-Asp, Pen and DNAm (i.e., included CpG sites) in bone (cf. Figure 1). One limitation was that the Pen accumulation during life was below the detectable threshold for some individuals under the age of 18 years.

Age-dependence was verified in bone with ρ > 0.8 in all markers, however, with a lower age-dependent correlation for D-Asp (ρ = 0.86) compared to previous studies using highly bradytrophic and homogeneous tissues, such as dentine (Pearson *r* = 0.96–0.99 [9, 12, 13, 49]). The age-dependent correlation for Pen was comparable to results for dentine (Pearson *r* = 0.94 [12, 15]).

Differences in the results for the protein parameters in total dentine and total bone can be explained by very different turnover rates in these tissues. Although dentine and bone share structural and functional similarities like the collagen matrix and the mineral content, after initial formation during tooth development, mature dentine is a very bradytrophic tissue with (almost) no turnover through which its protein composition stays largely unchanged [50] resulting in a close relation between D-Asp and Pen levels to age [12] even by analyzing total tissue.

Bone tissue on the other hand undergoes constant remodeling through a balanced process of old bone resorption and new bone replacement described as bone turnover rate (% rebuilt bone per year) and is influenced by e.g., diseases, stress, overall fitness, and hormonal influences [51]. It depends on bone type and is highest at sites where trabecular bone predominates and lowest at sites with a lot of cortical bone [4, 5]. In our study, we investigated skull bone samples having a higher density of cortical bone material and therefore a lower turnover rate [5]. However, with increasing age, the bone structure and metabolism change, resulting in loss of bone mass, decreasing thickness and osteoporosis [16, 18, 26]. It can therefore be assumed that the composition of the organic bone protein matrix varies, especially in old age, with each protein having its own kinetics for the accumulation of D-Asp and Pen, depending on its structure and metabolism. Consequently, variation in the protein composition through changes in bone metabolism as well as degradation of a total bone protein sample strongly impacts the D-Asp and Pen content of a total protein mixture. The only solution is the purification of individual long-living proteins. The analysis of D-Asp in purified osteocalcin from skull bones proves this theory with a very high correlation between D-Asp content in purified osteocalcin and age (Pearson *r* = 0.99 [52]). So far, only osteocalcin has been identified as a suitable bone protein; however, its purification is very challenging. The identification of further suitable proteins and the establishment of practicable methods for protein purification is an important research goal. We confirmed the results from our pilot study (D-Asp ρ = 0.9; Pen ρ = 0.9) investigating bone samples from 15 individuals [37]. It has to be mentioned that a comparison between studies is limited as sample ranges, age composition within the dataset, and the used correlation parameter can be discrepant between studies.

Also the observed Spearman’s correlation values for the DNAm markers (ρ = 0.87–0.93) were within the ranges of the pilot study (ρ = 0.9–0.93 [37]), although, the final ‘best’ CpG site was not the same in all cases. It would also be possible, to use neighboring CpG sites as an alternative, as these often had similar correlation values. Slight fluctuations can be caused by sample size and age composition under study. Other studies have also analyzed DNAm in bone samples and revealed age-dependent changes [22, 38, 39, 53, 54]. Furthermore, the six genomic regions show agedependent correlation in a wide range of other tissues and are (with different intensity) implemented in multiple age predictions models [30]. Although DNAm was a more accurate marker for age prediction, inter-individual variation increased with age, and outliers occurred. The observed age dependence may (partly) also be explained by changes in metabolism and turnover with increasing age caused by possible shifts in cell-type composition and cell function in dependence on the above-mentioned factors.

### Age prediction based on three biological age estimators

For the development of the RR models, only samples of the collected individuals equal to and above 18 years were used as the training dataset (*n* = 86). Furthermore, two independent test datasets (individuals without signs of decomposition: *n* = 44, individuals with signs of decomposition: *n* = 48) were used to test the RR models. The RR models based on Pen and D-Asp in total protein samples resulted for the training data using CV in a mean MAE/ mean RMSE of 9.66 years/ 11.52 years (Pen), and 11.91 years/ 14.57 years (D-Asp). The results for D-Asp and Pen do not measure up compared to the data for dentine and purified osteocalcin from skull bone [12, 15, 52]. However, the known methodological approaches for purifying osteocalcin are very complex and can currently hardly be used in forensic practice. Given this context, total protein samples were analyzed here. The DNAm approach led to a lower mean MAE mean RMSE of 4.95 years/ 6.89 years and was therefore more accurate compared to the protein-based parameters (∆MAE of 5 years and 7 years). These results were confirmed by the independent test set (cf. Table 1). In a previous study based on dentine, age prediction models were developed that led to MAEs of 2.93 years for D-Asp and 3.41 years for Pen [12]. First, DNAm age prediction models were developed, having e.g., in the study of Woźniak et al. (2021), an MAE of 3.3 years and 3.4 years in the training and test dataset by analysis of occipital and femoral bone material [38]. Differences in the MAEs compared to previous studies may be partly due to increased ages included in our study. The studies mentioned before included samples from individuals under 80 years (with the exception of one training sample in the study of Woźniak et al. (2021)) [22, 38, 39, 53, 54]. In our study, all predictions models led to an increased MAE and RMSE in the older age groups, with a particularly strong decreased accuracy in the 80 + year’s age category (cf. Suppl. Table S3). As in case of other age prediction models based on molecular markers, the higher uncertainty in case of older individuals should be considered. For better interpretation of the obtained results, reporting of age group-dependent model evaluations parameters as presented in Suppl. Table S3 can be therefor helpful. In addition, information as the percentage of the correct predictions within a case-dependent useful interval (e.g. 61.4% +/- 5 years) could be added.

### Advantage of using combined models

The combination of Pen and D-Asp for development of a RR model increased the overall accuracy (training set CV: MAE 8.55 years, RMSE 10.18 years, test set: MAE 7.16 years, RMSE 9.16 years). The usefulness of this approach has already been demonstrated by combining the D-Asp and Pen content for age prediction in dentine obtaining a decrease in MAEs from 2.93 (D-Asp) and 3.41 years (Pen) to 2.68 years (combined) [12], observing the same effect for more complex tissues such as intervertebral discs and epiglottis [33]. Considering the single-molecular clock models, the accuracy (evaluated as MAE/RMSE) of the DNAm-based model was superior to that of the protein-based age prediction in total protein samples. Combining the DNAm with either D-Asp, Pen, or D-Asp and Pen did not show an improvement in overall accuracy considering individuals without signs of decomposition. Nevertheless, this does not exclude an improvement in single cases as the MAE and RMSE evaluate the model performance based on all test data results. Therefore, the conclusion that isolated DNAm would always be sufficient in specific individual cases could be too short-sighted. The inclusion of the protein levels (as well as the inclusion of DNAm in protein models) might be useful in order to outbalance influences like lifestyle, health status, and numerous diseases [14, 31, 32]. Further research is needed to investigate the not yet well understood impact of these different factors on chronological age prediction models to define guidelines for in which cases a combination might be (dis) advantageous.

### Impact of post-mortem changes on age prediction

In a next step, we examined samples with early to severe signs of decomposition and the effect on the prediction accuracies. In our study, all three molecular clocks were successfully analyzed. However, with even longer postmortem intervals, reliable and accurate DNAm may be difficult. Bone proteins may be quite well preserved for a long time [55–57]. Nevertheless, postmortem degradation of proteins that change the overall composition of total bone samples may be a problem, if total bone samples (and not defined, purified proteins) are analyzed. In dentine, Pen could be stable over very long PMIs up to thousands of years (at least in dentine), which would enable a wide application range of age estimation based on this parameter also in the anthropological-archaeological context [58]. It remains to be clarified whether this also applies to bones.

For all parameters, a moderate correlation with age was observed (ρ(Pen) = 0.68, ρ(D-Asp) = 0.59, ρ(DNAm(6CpGs)) = 0.4–0.73), which was lower compared to the samples from individuals without signs of decomposition (cf. Figures 1 and 3). The biggest differences were observed for the markers with the highest correlation value (ρ) in individuals without signs of decomposition: Pen (∆ρ 0.22), ELOVL2 (∆ρ 0.21), PDE4C (∆ρ 0.28), TRIM59 (∆ρ 0.34), and KLF14 (∆ρ 0.39). The results can mainly be attributed to increased variation of single samples. This variation is also visible for the other markers, but ha less impact on the correlation value (ρ) due to an already higher variation in individuals without signs of decomposition. More research is needed to explicitly determine the underlying biological and technical causes (of which some are discussed below). An overall lower accuracy with MAEs of 11.77 years (RMSE 15.07 years) for Pen and 11.68 years (RMSE 15.42 years) for D-Asp was obtained. The DNAm model still performed better with an MAE of 7.38 years (RMSE 10.39 years) compared to the protein-based parameters but less accurate than testing bones without signs of decomposition. A slight improvement was obtained for the RMSE (10.39 years (DNAm) vs. 9.08 years (combined)) by the combination of the three molecular clocks (cf. Table 1). The slightly greater drop of the RMSE compared to the MAE (7.38 years (DNAm) vs. 6.8 years (combined)) may give an indication that especially outliers in the age prediction were reduced, which was the case in samples with very low DNA content (cf. Suppl. Figure 4C). An analysis of more samples is needed to support this indication.

The overall reduced accuracy in the age prediction based on the molecular clock models in decomposed individuals could be caused by postmortem changes like deterioration of the mineral phase and microbiological invasion which results in chemical and biological degradation of the organic bone matrix. In the absence of functional enzymatic repair mechanisms cellular components and DNA degrade due to their limited chemical stability [59, 60]. This leads to a change of the cell type composition and the amount and quality of DNA available for DNAm analysis.

Although bone proteins may be quite well preserved for a long time [55–57], postmortem degradation of proteins may significantly change the overall composition of total bone samples to a mixture of preserved proteins and fragments of broken proteins. This has direct implications on the overall contents of D-Asp and Pen, since they are analyzed as “summary values” in total protein samples. The even higher scattering of the data for older individuals could be related to a pre-existing intravital, age-related degradation of the organic bone matrix, which could result in a higher vulnerability against postmortem influences.

Additionally, the overall impaired tissue and cell structure in decomposed samples might have an impact. Especially for DNAm, a difference in the obtained DNAm values between individuals with and without signs of decomposition might occur due to the analysis process. As decalcification and multiple washing steps are part of the analysis, therefore a destroyed or altered cell structure could lead to a specific ‘wash away’ effect changing the cell type composition analyzed in the final eluate. Moving forward, extensive research is needed in the future to investigate the impact of the discussed degradation processes potentially interfering with accurate age prediction.

To get first research insight, if it could be beneficial to include the very heterogeneous biological as well technical variation caused by decomposition in the training data of a model, pilot RR models were built for age prediction of individuals with signs of decomposition. Within this model, the d-score was not yet included, as not enough samples covered all d-scores in sufficient amount. The overall visible state of decomposition of the body (total body d-score) and head (head d-score (cf. Suppl. Table S1B)) do not necessarily align with the decomposition state of specific tissues such as the bone material itself. As observed before, no correlation was seen between the dscore and the age prediction deviation (Suppl. Fig. S2). Overall, the pilot LOOCV RR models improved the prediction accuracy and outbalanced the previously observed downward trend (cf. Figure 4),but should be considered with caution as more research and samples are needed for a better understanding of all influences and to build a reliable model.

### Considerations and limitations of the developed models

The developed models are based on the results of the analyzed samples and might be influenced by that. Next to the biological facts impacting the results, the used technical procedures can lead to variation, limits, and to study-specific results which are presented below.

### Sample collection and preparation

Although the samples in this study were taken from strictly standardized areas (*Os Parietale*) at the same anatomical location, there could be differences and some heterogeneity between the cancellous and cortical portions within a bone fragment analyzed [61]. An additional factor causing variations in the proportion of cortical and cancellous bone is aging itself as described above [16]. This raises the question of whether different bone pieces from the same general location show intra-individual differences. Furthermore, variation between measurements even from the same fragment can occur because of stochastic effects (molecules analyzed) and technical fluctuations, which should be part of future research. Furthermore, our results cannot be automatically transferred to other bone types analyzed (e.g. femur, an often-occurring sample type in forensic casework). A study by König et al. (2023) observed differences in the age-dependent accumulation of D-Asp and Pen between three bone types (skull, rib, clavicle), which could be due to differences in the structure and metabolism of the various bone types from different anatomical regions, leading to different protein compositions and thus to variations in D-Asp and Pen levels of the samples [62]. The resulting impact on age prediction models has to be further investigated and will also depend on the strategy of the model development (e.g. choice of mathematical model, inclusion of sampling location).

As the analysis of all molecular markers depends on sample preparation, the use of another sample preparation before analysis could lead to differences. As described above, e.g. longer incubation steps for bone decalcification or increased wash steps prior to DNAm analysis might lead to ‘wash-away’ effects and change cell type composition. Furthermore, blood cells in small capillaries and the remaining bone marrow cannot be excluded as trabecular bone was not removed.

### Technical challenges

The data used for model development are based on D-Asp, Pen and DNAm analysis and technical variation has to be considered. Within this study, standardized methods were used for all samples to harmonize analysis over all three datasets. Technical variation was reduced to a minimum by using enough material e.g. to allow a DNA input of at least 10 ng in the PCR, reducing stochastic variation. Nevertheless, as shown especially for the decomposed individuals, that was not always possible. Furthermore, the harsh process of bisulfite conversion increases DNA degradation with a higher impact on DNAm analysis in case of already pre-degraded samples. These effects remain a challenge in case of DNAm analysis from degraded and low DNA amounts increasing stochastic variation during DNAm analysis.

In case of protein analysis, technical variation can also arise by a too low powder amount (optimal amount in our study was identified with 20 mg) for a sufficient signal quantity for evaluation. Furthermore, the technical threshold for detection of the protein accumulation resulted e.g. in detection challenges for the Pen accumulation in minors. More sensitive methods might be help in the future to overcome that problem.

### Model development and evaluation

The presented models and results are based on the choice of six CpG sites, two protein markers and ridge regression as underlying mathematical model. The included CpG sites showed age-dependency, and the mathematical model showed suitability, however the use or addition of other sites and optimization of the mathematical model might be able to improve the model.

The models developed during this study are based on samples from deceased individuals and therefore limited in material available, leading to a model excluding individuals under 18 years of age. This decision was made to exclude a bias due to imbalance of number of individuals per age category and in addition due to the fact, that the interpretational threshold did not allow a reliable quantification of the Pen amount for all individuals under the age of 18 years. Within the study, sex balance between females and males as well as equal balance over the whole age group could not be completely achieved. A potential impact of the sex needs further consideration and deeper analysis with more samples. Furthermore, the composition of dataset 3 including individuals with signs of decomposition is biased toward a higher age due to the circumstance that younger individuals are less often found in a (highly) decomposed state. Further collection of samples of specific ages and decomposition state could help to improve model building in the future.

## Conclusion

In summary, molecular age prediction on skull bone is possible across all parameters examined. The DNAm-only model (6 CpGs) provided the best results. The protein-based parameters D-Asp and Pen yield (in the here analyzed total protein samples) weaker results than DNAm using univariate models; however, it should be noted that the “single” DNAm molecular clock model is based on 6 CpGs. An approach to more reliable results based on protein parameters could be the implementation of protein purification; the development of viable methods for protein purification could enable significantly more precise age diagnoses. By combining the parameters in postmortem age prediction in which decomposition results in an additional layer of variability biological material, age prediction models including multiple molecular clocks could provide improved accuracy due to the inclusion of independent parameters. Further research is needed to investigate in depth if the combination can compensate internal and external factors that influence single molecular clocks.

## Electronic supplementary material

Below is the link to the electronic supplementary material.

Supplementary Material 1

Supplementary Material 2

Supplementary Material 3

Supplementary Material 4
