# Supplementary material for: Molecular age prediction using skull bone samples from individuals with and without signs of decomposition: a multivariate approach combining analysis of posttranslational protein modifications and DNA methylation
Source: Int J Legal Med. 2024 Sep 11;139(1):157–74. doi: 10.1007/s00414-024-03314-z (PMC11732915; doi:10.1007/s00414-024-03314-z)
Supplement: Supplementary file 3 — Supplementary Material 3 [file 414_2024_3314_MOESM3_ESM.pdf]

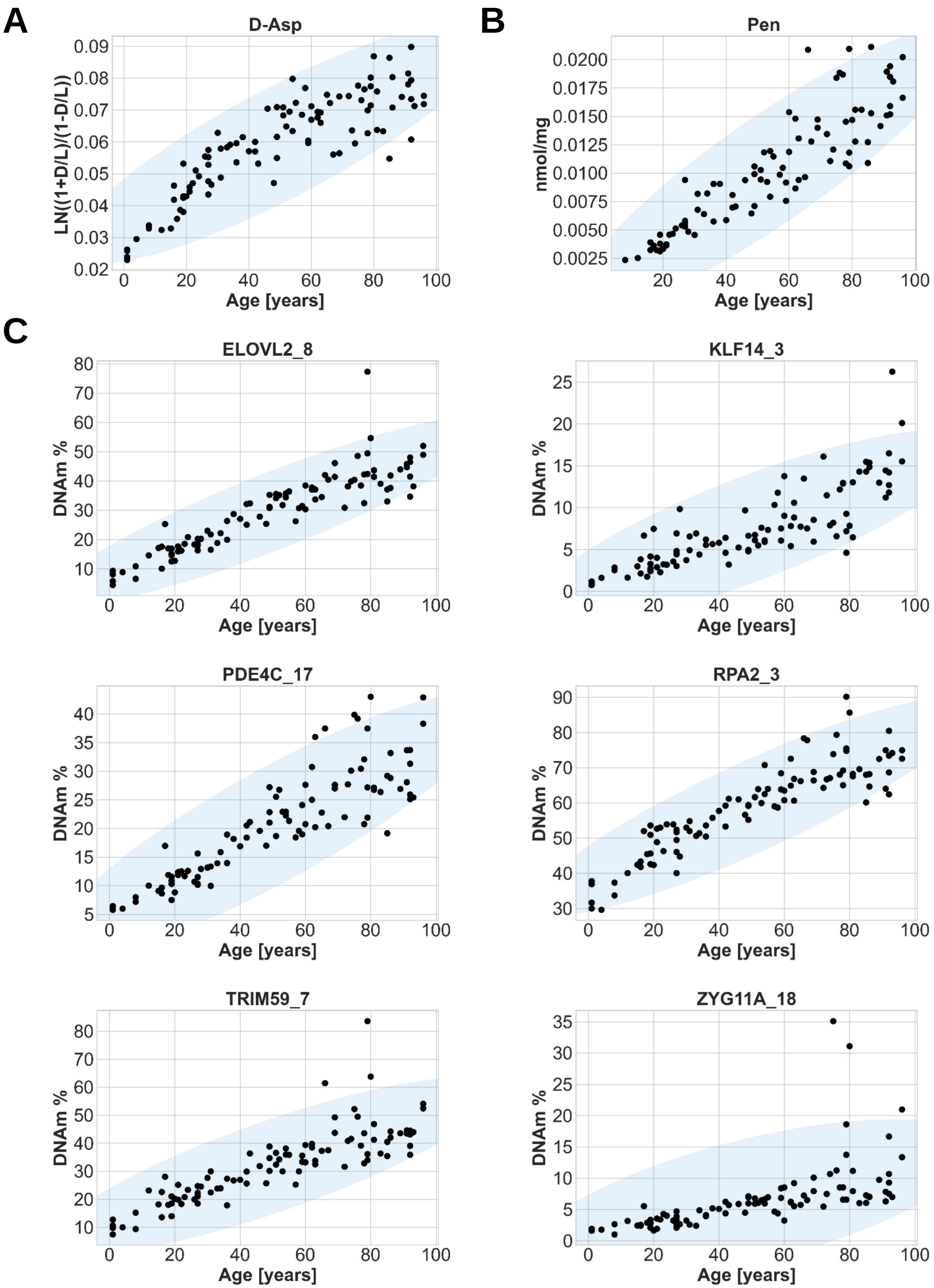

Fig S1. Malahanobsis analysis. Values outside the blue ellipse were identified as outlier samples. A) Accumulation of D-Asp and Pen. In case of Pen, values are missing due to detection limits for most of the under 15 years old. B) DNA methylation levels for the final selected CpG sites from six amplicon regions. ρ: Spearman‘s rho. D: D-Asp, L: L-Asp; n=98.

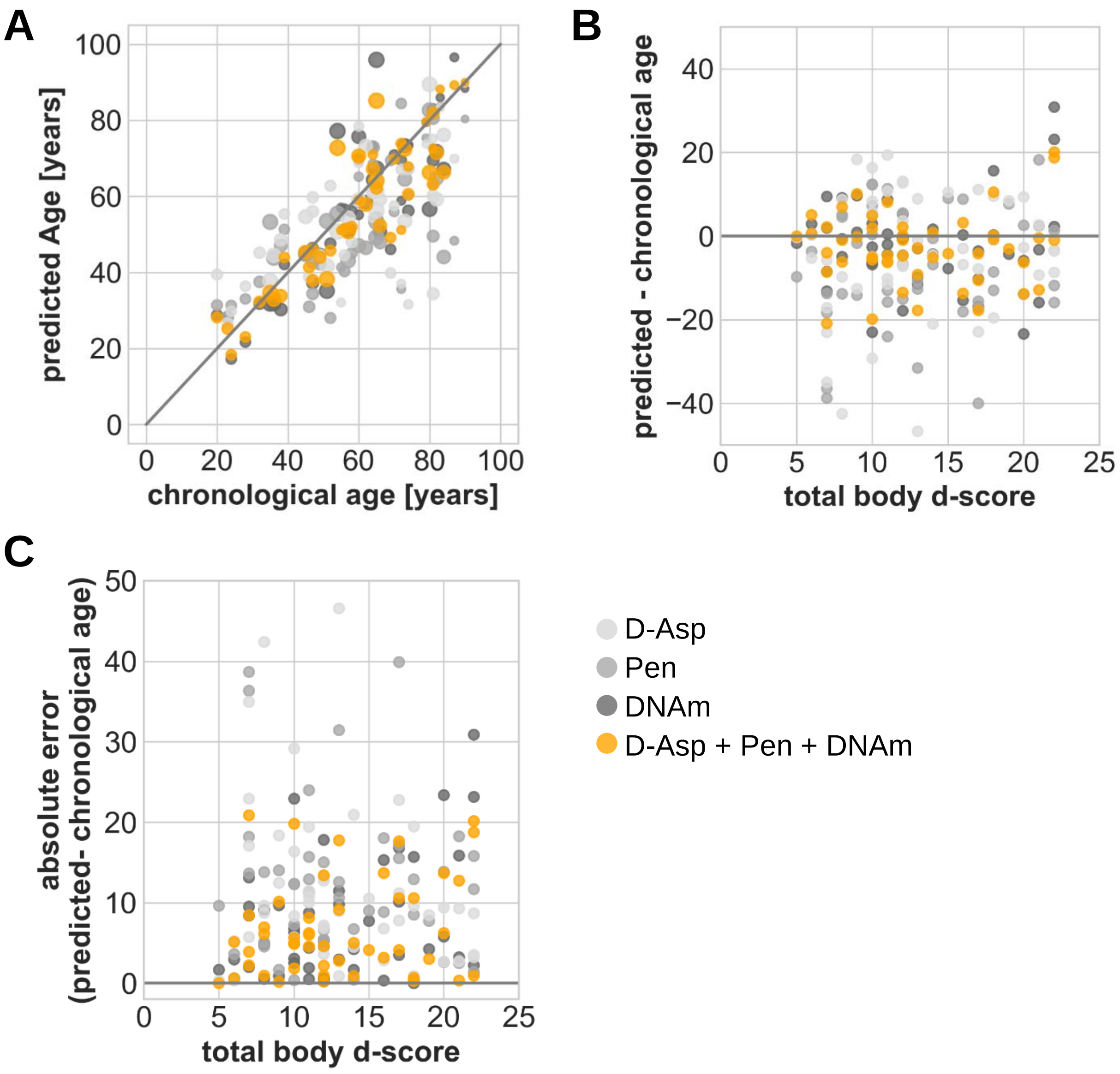

Fig. S2 Deeper investigation of samples from decomposed individuals
Age predictions (A), deviations (B) and absolute deviations (C) using the single-molecular clocks RR models for DNAm (dark grey), Pen (grey), D-Asp (lightgrey), and combined (DNAm, Pen, D-Asp) (orange) trained with the individuals without signs of decomposition. Marker size in A represents the total body p-score.

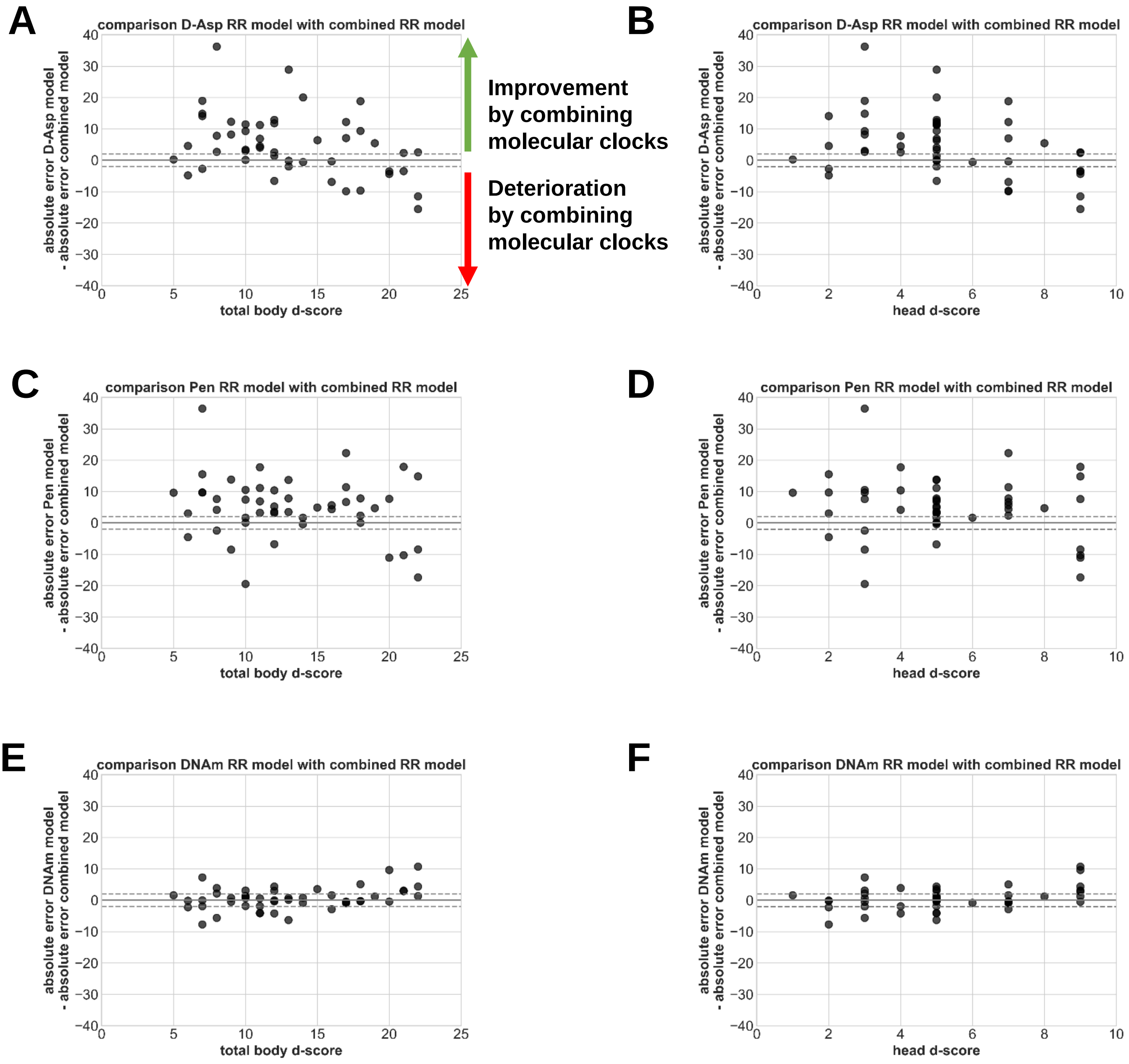

Fig. S3 Deeper investigation of samples from decomposed individuals
Difference of absolute prediction error for the single-molecular clock models and the combined model. Values above 2 indicate an improvement, values below -2 a decrease in accuracy. Results are presented in dependence to total body p-score and head p-score for D-Asp – combined (A, B), Pen – combined (C, D), and DNAm – combined (E, F)

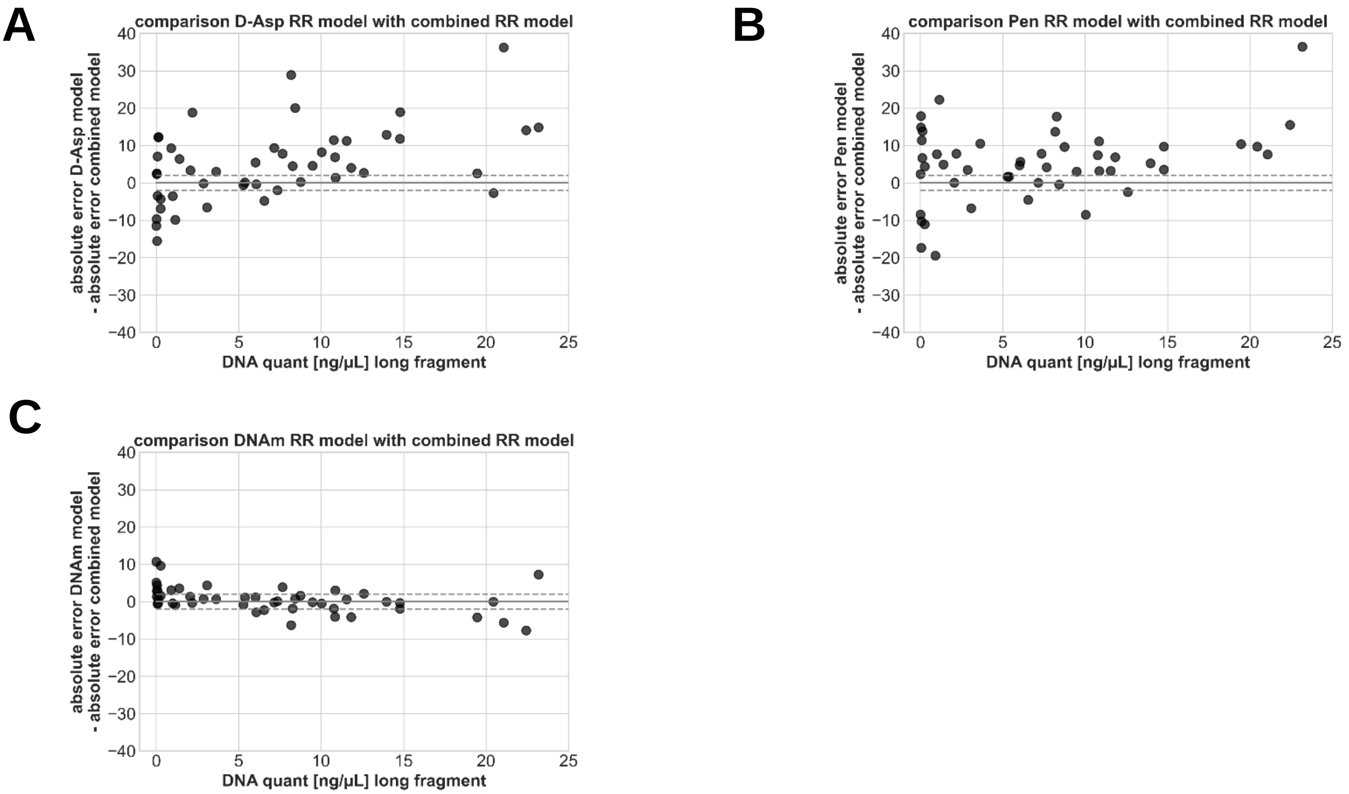

Fig. S4 Deeper investigation of samples from decomposed individuals
Difference of absolute prediction error for the single-molecular clock models and the combined model. Values above 2 indicate an improvement, values below -2 a decrease in accuracy. Results are presented in dependence of the DNA quantity (determined on the longer fragment in the PowerQuant quantification system) for D-Asp – combined (A), Pen – combined (B), DNAm – combined (C)
