# Supplementary material for: Molecular age prediction using skull bone samples from individuals with and without signs of decomposition: a multivariate approach combining analysis of posttranslational protein modifications and DNA methylation
Source: Int J Legal Med. 2024 Sep 11;139(1):157–74. doi: 10.1007/s00414-024-03314-z (PMC11732915; doi:10.1007/s00414-024-03314-z)
Supplement: Supplementary file 4 — Supplementary Material 4 [file 414_2024_3314_MOESM4_ESM.pdf]

Supplementary Table S3: Evaluation of age predictions dependent on age groups. As the training data set was evaluated using 10 times 10xfold CV, the mean CV over all 10 repetitions is provided.
CV= cross valiadation, LOOCV= leave-one-out CV, MAE= mean absolute error, RMSE= root mean square error, DNAm= DNA methylation, D-Asp= accumulation of D-aspartic acid, Pen= accumulation of pentosidine, y= years

Age group	Dataset	Evaluation parameter	DNAm	D-Asp	Pen	Asp + Pen	DNAm + Asp + Pen
Training data (CV) (n=86)	1 (18 y-39 y) n=26	mean MAE	2.76	10.81	9.01	6.26	2.36
		mean RMSE	3.75	12.78	10.06	7.52	3.07
	2 (40 y-59 y) n=20	mean MAE	2.61	11.09	5.59	6.64	3.17
		mean RMSE	3.19	12.98	6.67	7.87	3.84
	3 (60 y-79 y) n=22	mean MAE	5.65	8.30	12.28	10.64	6.48
		mean RMSE	8.71	11.75	14.66	12.46	9.03
	4 (80 y +) n=18	mean MAE	9.67	18.92	11.78	11.41	8.71
		mean RMSE	11.33	22.02	14.03	13.40	10.29
Test data (n=44)	1 (18 y-39 y) n=11	MAE	4.93	9.41	5.96	5.03	4.28
		RMSE	5.56	11.42	7.30	6.80	5.08
	2 (40 y-59 y) n=11	MAE	4.06	10.26	8.32	6.78	4.62
		RMSE	4.50	11.44	9.00	8.49	5.22
	3 (60 y-79 y) n=15)	MAE	6.58	11.44	7.30	8.25	5.53
		RMSE	7.56	13.89	8.67	9.58	6.89
	4 (80 y +) n=7	MAE	6.20	18.24	10.46	8.75	6.26
		RMSE	8.22	23.20	12.91	12.03	8.22
Decomposed data (n=48)	1 (18 y-39 y) n=9	MAE	5.07	9.83	9.10	6.65	3.75
		RMSE	5.66	11.19	9.97	7.87	4.46
	2 (40 y-59 y) n=12	MAE	5.75	9.47	10.28	9.38	6.55
		RMSE	8.97	10.76	12.35	11.36	8.15
	3 (60 y-79 y) n=18	MAE	8.53	12.63	11.16	10.95	7.63
		RMSE	12.22	17.14	13.67	14.03	10.40
	4 (80 y +) n=9	MAE	9.57	14.57	17.66	16.60	8.50
		RMSE	11.74	20.02	23.12	20.73	10.71
LOOCV-decomposed data (n=48)	1 (18 y-39 y) n=9	MAE	5.51	20.04	20.20	17.42	5.72
		RMSE	6.96	20.49	20.43	17.66	6.82
	2 (40 y-59 y) n=12	MAE	4.38	7.16	5.61	5.60	4.39
		RMSE	5.95	9.04	6.34	6.34	5.82
	3 (60 y-79 y) n=18	MAE	7.49	9.71	7.87	8.14	6.78
		RMSE	8.84	13.00	9.43	10.88	8.06
	4 (80 y +) n=9	MAE	8.66	14.65	14.59	14.32	7.87
		RMSE	9.84	17.72	18.65	17.56	9.33
